# Standardized Workflow for Precise Mid- and High-Throughput Proteomics of Blood Biofluids

**DOI:** 10.1093/clinchem/hvab202

**Published:** 2022-03-04

**Authors:** Angela Mc Ardle, Aleksandra Binek, Annie Moradian, Blandine Chazarin Orgel, Alejandro Rivas, Kirstin E. Washington, Conor Phebus, Danica-Mae Manalo, James Go, Vidya Venkatraman, Casey W. Coutelin Johnson, Qin Fu, Susan Cheng, Koen Raedschelders, Justyna Fert-Bober, Stephen R. Pennington, Christopher I. Murray, Jennifer E. Van Eyk

**Affiliations:** a Smidt Heart Institute, Advanced Clinical Biosystems Research Institute, Cedars-Sinai Medical Center, Los Angeles, CA, USA; b Precision Biomarker Laboratories, Cedars-Sinai Medical Center, Los Angeles, CA, USA; c Smidt Heart Institute, Barbra Streisand Women’s Heart Center, Cedars-Sinai Medical Center, Los Angeles, CA, USA; d School of Medicine and Medical Sciences, UCD Conway Institute of Biomolecular and Biomedical Research, University College Dublin, Dublin 4, Ireland

## Abstract

**BACKGROUND:**

Accurate discovery assay workflows are critical for identifying authentic circulating protein biomarkers in diverse blood matrices. Maximizing the commonalities in the proteomic workflows between different biofluids simplifies the approach and increases the likelihood for reproducibility. We developed a workflow that can accommodate 3 blood-based proteomes: naive plasma, depleted plasma and dried blood.

**METHODS:**

Optimal conditions for sample preparation and data independent acquisition-mass spectrometry analysis were established in plasma then automated for depleted plasma and dried blood. The mass spectrometry workflow was modified to facilitate sensitive high-throughput analysis or deeper profiling with mid-throughput analysis. Analytical performance was evaluated by the linear response of peptides and proteins to a 6- or 7-point dilution curve and the reproducibility of the relative peptide and protein intensity for 5 digestion replicates per day on 3 different days for each biofluid.

**RESULTS:**

Using the high-throughput workflow, 74% (plasma), 93% (depleted), and 87% (dried blood) displayed an inter-day CV <30%. The mid-throughput workflow had 67% (plasma), 90% (depleted), and 78% (dried blood) of peptides display an inter-day CV <30%. Lower limits of detection and quantification were determined for peptides and proteins observed in each biofluid and workflow. Based on each protein and peptide’s analytical performance, we could describe the observable, reliable, reproducible, and quantifiable proteomes for each biofluid and workflow.

**CONCLUSION:**

The standardized workflows established here allows for reproducible and quantifiable detection of proteins covering a broad dynamic range. We envisage that implementation of this standard workflow should simplify discovery approaches and facilitate the translation of candidate markers into clinical use.

## Introduction

Mass spectrometry (MS)-based proteomic analyses of blood provide a survey of an individual’s biological state (1). These insights can lead to an understanding of pathology and guide the development of clinically relevant biomarkers with diagnostic and prognostic utility for disease outcome and the response to treatment (1–3). The minimal invasiveness of sampling and the rich array of informative biomolecules make blood and its constituent fractions (plasma and serum) a routine biofluid for clinical testing and in biomarker discovery studies. More recently, dried blood spots and volumetric absorptive microsampling have emerged as alternative platforms for sample collection (4, 5). Dried blood can be obtained by an individual at home without medical expertise or refrigeration, greatly expanding the potential for remote and longitudinal sampling, and for aiding analyses in difficult to access populations. While diversity in sample collection presents new opportunities for discovery and validation studies, it also underscores the need for an adaptable, scalable MS-based analysis platform that provides reproducible and quantitative proteomic characterization that can facilitate the translation of new biomarkers to clinical use.

Common among the different blood biofluids is the dynamic complexity of their constituent proteomes. This complexity presents substantial obstacles for robust proteomic analyses. Liquid chromatography–mass spectrometry (LC-MS) can become overwhelmed by the highly abundant resident blood proteins and can fail to detect those less-abundant proteins that may signify a change in health status. Two archetypical strategies have been developed to overcome this challenge: (a) simplify the analyte by removing components that are unlikely to provide diagnostic value, and (b) optimize detection to make the survey of the biofluid more comprehensive. For example, simplifying blood to plasma or serum removes the cellular and platelet components; however, a small number of highly abundant proteins still dominate the analysis (6). These biofluids can be further simplified using selective depletion methods such as the 14 most abundant proteins in plasma/serum (7) or targeted enrichment methods (8–10). In practice, depleted plasma is not simply a subset of naive plasma. The change in protein composition fundamentally alters the analytical characteristics of the matrix establishing a distinct biofluid where unique components of the blood proteome can be observed. As such, the two biofluids can be evaluated in parallel, and the analysis combined for a more complete evaluation of the original sample.

A common method to evaluate a proteome by mass spectrometry is data independent acquisition-MS (DIA-MS) (11). In DIA-MS, peptides that co-elute from the liquid chromatography (LC) column are systematically fragmented into a series of overlapping mass windows. The fragmentation process is repeated for the duration the chromatographic separation producing a comprehensive survey of peptides in a sample. MS instrument parameters such as the size of the precursor mass windows or the resolution can be altered to improve protein depth and analytical precision in a DIA-MS method (12). The choice of LC column and the length of separation gradient can each alter the number of peptides detected and their resolution. A central dilemma in biomarker discovery is achieving a balance between greater depth of proteome coverage and the throughput needs of a large discovery or validation cohort while maintaining analytic precision. In this study we have focused on 2 levels of sample throughput: a high-throughput method (21-min gradient; 25 min/sample) where 57 samples can be run in 24 h or a mid-throughput method (60-min gradient; 72 min/sample) where 20 samples can be run in 24 h. These two methods provide representative options for contrasting depth and speed of an analysis.

**Fig. 1.**
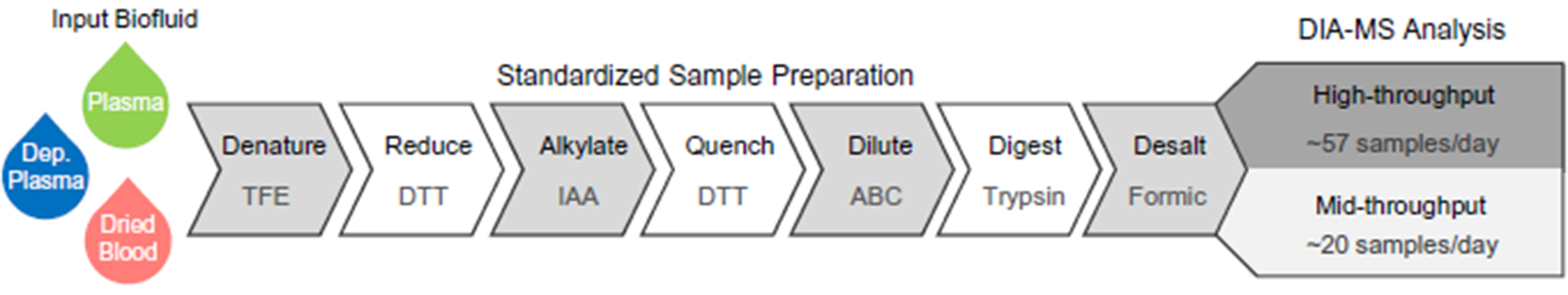
Standardized workflow for proteomic analysis of blood biofluids.

With these challenges in mind, we set out to establish a streamlined preanalytical proteomic workflow that balances sample matrix, proteome depth, throughput, and quantifiability in one unified proteomic platform (Fig. 1). A robust standardized MS workflow that can accommodate multiple inputs while achieving analytical precision is a necessity to develop translatable clinical biomarkers.

## Materials and Methods

### TEST BLOOD BIOFLUID SAMPLES

Commercially available pooled mixed sex plasma (K_2_EDTA) was used for both analysis of naive and depleted plasma. Whole blood (K_2_EDTA) was purchased from Bioreclamation and mock collected using Mitra^®^ microsampling devices (Neoteryx). Further details can be found in the online Supplemental Information.

### 96-WELL FORMAT FOR DEPLETION OF ABUNDANT PLASMA PROTEINS PROTEIN DEPLETION FOR PLASMA

Plasma samples were depleted of the 14 most abundant proteins including albumin, immunoglobulins A, E, G, and M, kappa and lambda light chains, alpha-1-acidglycoprotein, alpha-1-antitrypsin, alpha-2-macroglobulin, apolipoprotein A1, fibrinogen, haptoglobin, and transferrin using the High Select Top 14 Abundant Protein Depletion Camel Antibody Resin (Thermo Fisher Scientific), as described in the online Supplemental Information.

### AUTOMATED BLOOD BIOFLUID TRYPSIN DIGESTION AND DESALTING

All blood biofluids underwent protein denaturation, reduction, alkylation, digestion using the Beckman i7 automated workstation (Beckman Coulter) programed for uniform mixing as previously described at a controlled temperature and modified with online automated desalting (13). Proteins were denatured in a solution of 350 mL/L 2,2,2-trifluoroethanol (TFE, Sigma), 40 mM dithiothreitol (Sigma) and dissolved in 50 mM NH_4_CO_3_ (Sigma). The sample was denatured for 1 h at 60°C. Samples were then alkylated for 30 min at 25°C in the dark with the addition of iodoacetamide (Sigma; 10 mM final concentration). To prevent overalkylation, dithiothreitol was added at final concentration of 5 mM and samples were incubated for a further 15 min at 25°C. Next, a volume of 50 mM NH_4_CO_3_ was added to dilute out TFE to a final concentration of 50 or 100 mL/L. Trypsin was added to a ratio of 25:1 and samples were incubated for 4 or 16 h at 37 or 42°C. Digestion reactions were quenched with 5 μL of 250 mL/L formic acid. Desalting was carried out using a positive pressure apparatus (Amplius Positive Pressure ALP, Beckman Coulter) mounted on the left side of the i7 workstation deck. Further details are provided in the online Supplemental Information and Supplemental Fig. 1. Peptide concentrations for sample loading were based on a standard protein concentration of 50 μg/μL in plasma. After desalting, samples were diluted with 1 mL/L formic acid to the stock concentration of 1 μg/μL and further diluted to a desired concentration for MS analysis. The same relative concentration and dilution strategy was applied to depleted plasma and dried blood samples.

### HIGH- AND MID-THROUGHPUT DIA LC-MS/MS

DIA-MS analysis was performed on an Orbitrap Exploris 480 mass spectrometer (Thermo Scientific). The high-throughput workflow utilized the Evosep One system with a 21-min gradient and 4-min wash step, requiring 25 min to complete each sample. The mid-throughput workflow was run on an Ultimate 3000 ultra high-pressure chromatography system with a 60-min gradient and 12-min wash, and required 72 min to complete (see online Supplemental Information). For the linearity experiments, serial dilutions of each biofluid were prepared by adding 1 mL/L formic acid to desalted samples. The high-throughput method was evaluated using a 6-point curve (31 to 1000 ng on column) while the mid-throughput method had a 7-point range (39 to 2500 ng on column).

To determine reproducibility of the peptide intensities, 5 replicate samples of each biofluid were digested once a day for 3 consecutive days (for a total of 15 runs) and analyzed using the different workflows. In the high-throughput workflow, 250 ng of naive plasma, 125 ng of depleted plasma, or 250 ng of dried blood were injected on column and evaluated by MS. Since the longer separation gradient used in the mid-throughput workflow could accommodate a greater peptide load, 1250 ng of naive plasma, 625 ng of depleted plasma, or 2500 ng of dried blood were injected on column and evaluated by MS.

### BIOINFORMATIC DATA ANALYSIS

A full description of the data analysis and all peptide and proteins identifications including reproducibility and linearity characterizations can be found in the online Supplemental Information and Supplemental Tables 1 to 20 (accessible in the Panorama public repository, PXD ID: PXD024884) (14). In the linearity experiments, the lower limit of detection (LLOD) for a protein or peptide was determined by the lowest sample load where the unnormalized protein or peptide intensities were detected in 2 out of 3 replicates with a CV < 20%. The lower limit of quantification (LLOQ) for a protein or peptide was determined by the lowest sample load where the protein or peptide is detected in 2 out of 3 replicates with a square of the correlation coefficient (*r*^2^) > 0.8, CV < 20% and a target deviation >0.2.

In the reproducibility study, the CVs for MS2 total ion current (TIC) normalized protein or peptide intensities were determined if there were at least 3 out of 5 observations on each day. This threshold was required for all 3 days to determine a multi-day CV. The results for each biofluid and workflow were divided in to 4 categories: observable proteome (observed at least once in any run on any day); reliable proteome (observed at least 3 times on each of the 3 days); reproducible proteome (observed at least 3 times on each of the 3 days with a multi-day CV < 30%); quantifiable proteome (observed at least 3 times on each of the 3 days with a multi-day CV < 30% and an *r*^2^ > 0.8 determined from the linearity experiments) (Table 1).

## Results

### OPTIMIZATION OF DIGESTION AND DIA-MS PARAMETERS IN PLASMA

**Fig. 2.**
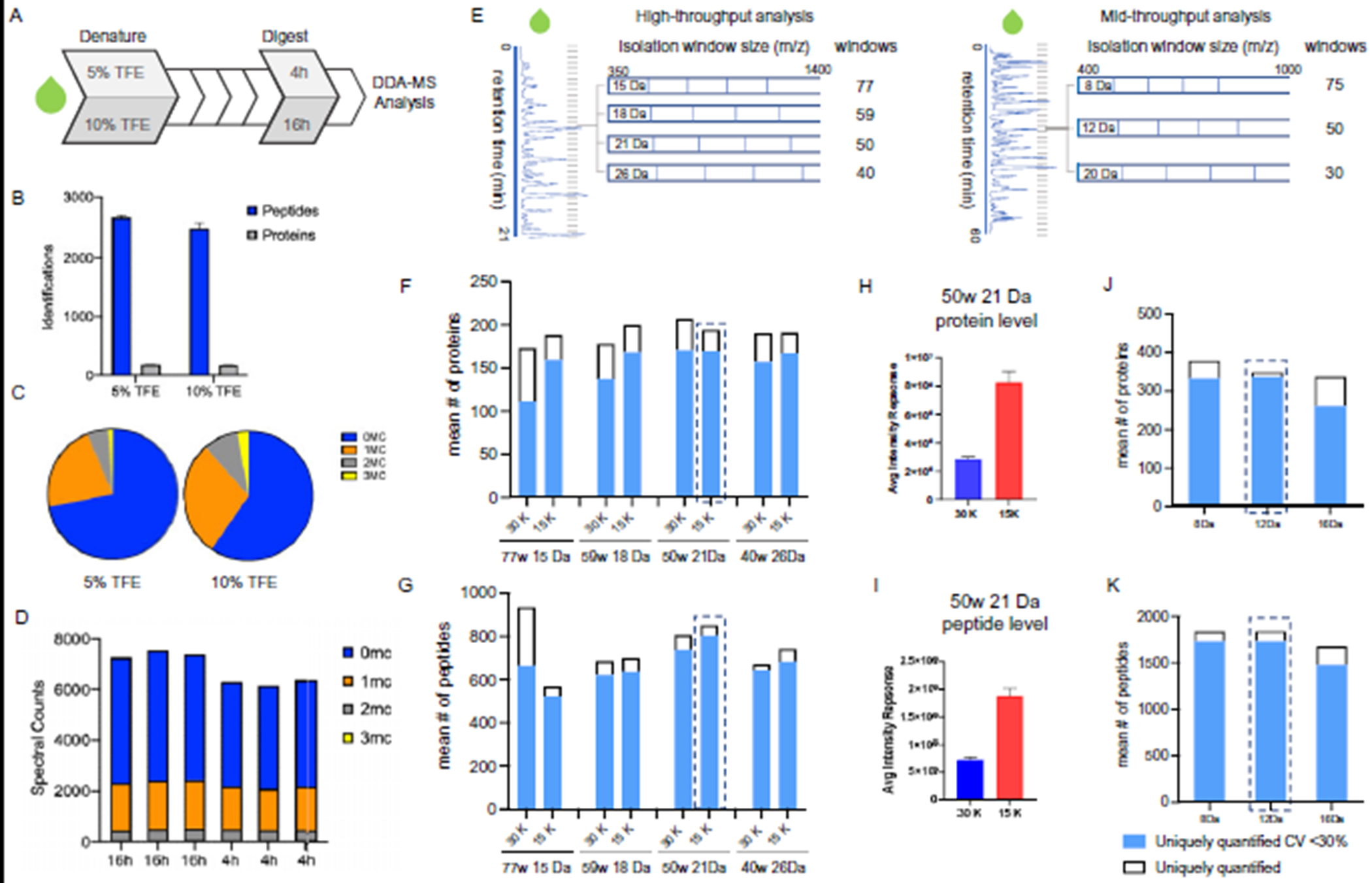
Optimization of digestion and DIA-MS parameters in naive plasma. (A) Scheme for the optimization of 2 parameters, denaturation conditions and proteolysis time as defined in Fig. 1. (B) Yield of peptide and protein identification under different denaturation buffers conditions (50 mL/L vs 100 mL/L TFE). (C) Number of peptides with 0, 1, 2 or 3 miscleavages (MC) recovered from digestion when using 50 mL/L or 10LL0 mL/L TFE denaturation buffers. Blue, orange, grey and yellow show proportion of peptides with 0, 1, 2, and 3 MC, respectively. (D) Spectral counts of peptides with 0, 1, 2 or 3 MC in each digestion replicate at 4 h and 16 h of incubation. (E) Overview of the DIA-MS optimization experiments. (F and G) The number of proteins and peptides uniquely identified and quantified with CV < 30% when analyzed with 8 different MS settings using the high-throughput workflow. (H and I) Mean intensity response of all proteins and peptides when analyzed with a resolution setting of 15 000 vs 30 000 (50 windows (w) 21 Da method). (J and K) The number of proteins and peptides uniquely identified and quantified with CV < 30% when analyzed with the mid-throughput workflow.

The volatile denaturant, TFE, was used to improve digestion efficacy (Fig 2, A); however, the final concentration can influence the pH of the digestion buffer and impact trypsin activity (15, 16). Plasma was selected as the representative biofluid in the optimization experiments for its ubiquity in discovery cohorts (17–19). Analysis of plasma digested in 50 mL/L or 100 mL/L TFE revealed that 50 mL/L TFE resulted in a marginal increased number of peptide identifications (2686) compared to 100 mL/L TFE (2502) (Fig. 2, B). This observation can be attributed to the decreased rate of peptide miscleavages, a surrogate measure for proteolytic efficiency (Fig. 2, C) (20). Reducing miscleavages is key as the signal loss from inconsistent digestion can lead to dubious relative intensity values.

We also assessed the impact of time on tryptic proteolytic efficiency (Fig. 2, A) using manual sample preparation. To reduce sample processing times, we evaluated the impact of trypsin incubation length. Plasma was digested for 4 or 16 h and analyzed by MS (n = 3). Using a 16 h incubation period yielded a slightly greater (0.8-fold) peptide intensity response vs 4 h (Fig. 2, D). Even though the 16 h digestion displayed more favorable data in terms of the number of identifications and relative intensity values, the duration of proteolysis did not impact on reproducibility and the time savings allowed sample processing to be completed in a single day. To maximize throughput and efficiency, we selected 4 h trypsinization in the presence of 5 mL/L TFE for inclusion into our final protocol.

With a streamlined set of preparation conditions, we used plasma to optimize the DIA-MS acquisition parameters. In DIA-MS, peptides that fall within a defined mass window are fragmented in the orbitrap and analyzed together. The instrument cycles through subsequent overlapping windows until the entire mass range is covered. The instrument cycle time is influenced by resolution settings and the number of mass windows, which in turn impact the number of identifications and reproducibility. Based on previous reports in plasma (21), we compared the performance of 250 ng of digested naive plasma in 8 MS methods covering 4 isolation window widths and 2 resolutions (15 Da, 77 windows; 18 Da, 54 windows; 21 Da, 50 windows; 26 Da, 40 windows; each at 15 000 or 30 000 MS2 resolution) in the high-throughput workflow (Fig. 2, E). The number of protein identifications across all parameters were comparable, ranging from a mean of 173 to 206 identifications (Fig. 2, F). The 15 Da, 77 isolation windows at 30 000 resolution method supported the most peptide identifications compared to the other settings assessed (Fig. 2, F and G). Despite being associated with the greatest number of peptide identifications, this method performed worst in terms of reproducibility (144 proteins, CV < 30%, n = 3). The 21 Da, 50 window method using 15 000 resolution was associated with the greatest number of quantifiable peptides (841) with CV < 30%.

Next, we investigated the impact of MS resolution settings, 15 000 or 30 000, on overall plasma protein and peptide intensity. We calculated the mean intensity response from all identified proteins and peptides analyzed with 21 Da method employing either 15 000 or 30 000 resolution and found that 15 000 yielded the greater intensity response (Fig. 2, H and I). We also observed lower CVs across the range of observed peptide intensities for 15 000 compared to 30 000 resolution (see online Supplemental Fig. 2, A to C). We hypothesize that this observation was attributed to the number of data points acquired across the curve for all detected peptides. In a comparison using the 11 iRT peptides, the 21 Da at 15 000 method performed second best in terms of the number data points collected (13.0 (2.7), n = 3) (see online Supplemental Fig. 2, D). Importantly, data acquired using this method were associated with >8 data points across the curve, a well-established benchmark for acceptable quantification (22).

Leveraging the results from the high-throughput workflow, we compared 3 DIA methods for our 72-min, mid-throughput workflow using 1250 ng of digested naive plasma. Since this platform supported longer peptide separation, we designed DIA methods with narrower isolation windows (8, 12 and 20 Da) to increase identifications (Fig. 2, E). Here the broadest window setup (20 Da) was associated with the lowest number of proteins (292) and peptides (1576) with CV < 30%. While the data indicated the 12 Da method performed best (325 proteins, 1800 peptides CV < 30%, n = 3) (Fig. 2, J and K). As before, we investigated the impact of isolation window settings on the number of data point across the curve with 12 Da performing second best (see online Supplemental Fig. 2, E). Taken together, the 12 Da method was selected as optimal (22).

### QUANTIFIABILITY OF 3 BIOFLUIDS USING STANDARD METHODS

**Fig. 3.**
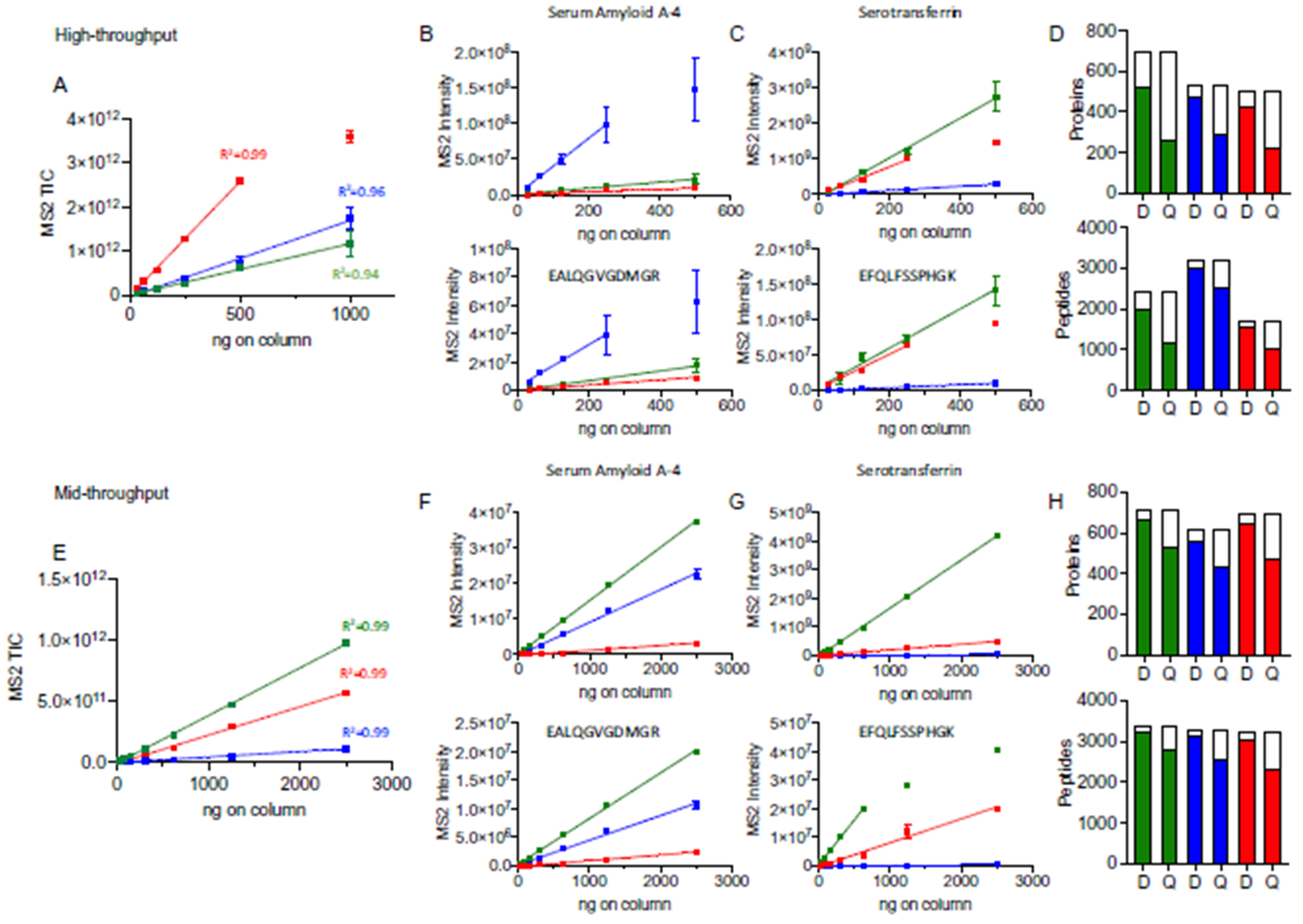
Comparison of linearity across biofluids and throughputs. Assessment of total MS2 signal intensity across increasing column load for naive plasma (green), depleted plasma (blue), and dried blood (red) using the (A) high- and (E) mid-throughput workflow. The range of linear response is shown for selected proteins, serum amyloid A-4 (B and F) and serotransferrin (C and G), and representative peptides. Panels (D) and (H) show a summary of the proteins and peptides with an observed LLOD or LLOQ in both throughput methods. Total column height indicates the number of proteins or peptides with 2 or more observations in at least one loading condition. The filled columns indicate the number of proteins or peptides where an LLOD or LLOQ (designated as D or Q) was determined. See Methods for a description of LLOD/Q determination.

With 2 optimal versions of the protocol established, we evaluated the quantifiability of our workflows on 3 commonly analyzed biofluids: naive plasma, depleted plasma, and dried blood. Serial dilution curves were prepared for each biofluid, and samples were analyzed using the high-throughput method (range: 31 to 1000 ng on column) and mid-throughput method (range: 39 to 2500 ng on column). Relative peptide quantities were determined using the MS2 TIC intensity across different loads (n = 3) (Fig. 3, A and E). It was possible to establish a linear response across 31 to 500 ng and 39 to 2500 ng for the high- and mid-throughput workflows, respectively, in plasma, depleted plasma and dried blood,. Examples of the linear response of serum amyloid A-4 (Fig 3, B and D) and serotransferrin (Fig. 3, C and G) are shown at the protein (upper panels) and representative peptide (lower panels) level for each biofluid and throughput method. Different relative responses were observed depending on the sample type and throughput method indicating differences in relative abundance and specific matrix effects. Using this analysis, an LLOD and LLOQ was determined for many of the peptides and proteins identified in the high- and mid-throughput analysis of plasma, depleted plasma and dried blood (Fig. 3, D and H). Overall, the mid-through put methods had a higher proportion of proteins and peptides where an LLOD or LLOQ was determined. Supplemental Tables 1 to 15, accessible from the Panorama public repository (PXD ID: PXD024884), provide a listing of the linear response, limits of detection, and quantifiability for each protein and peptide.

### PRECISION OF STANDARDIZED WORKFLOW

**Fig. 4.**
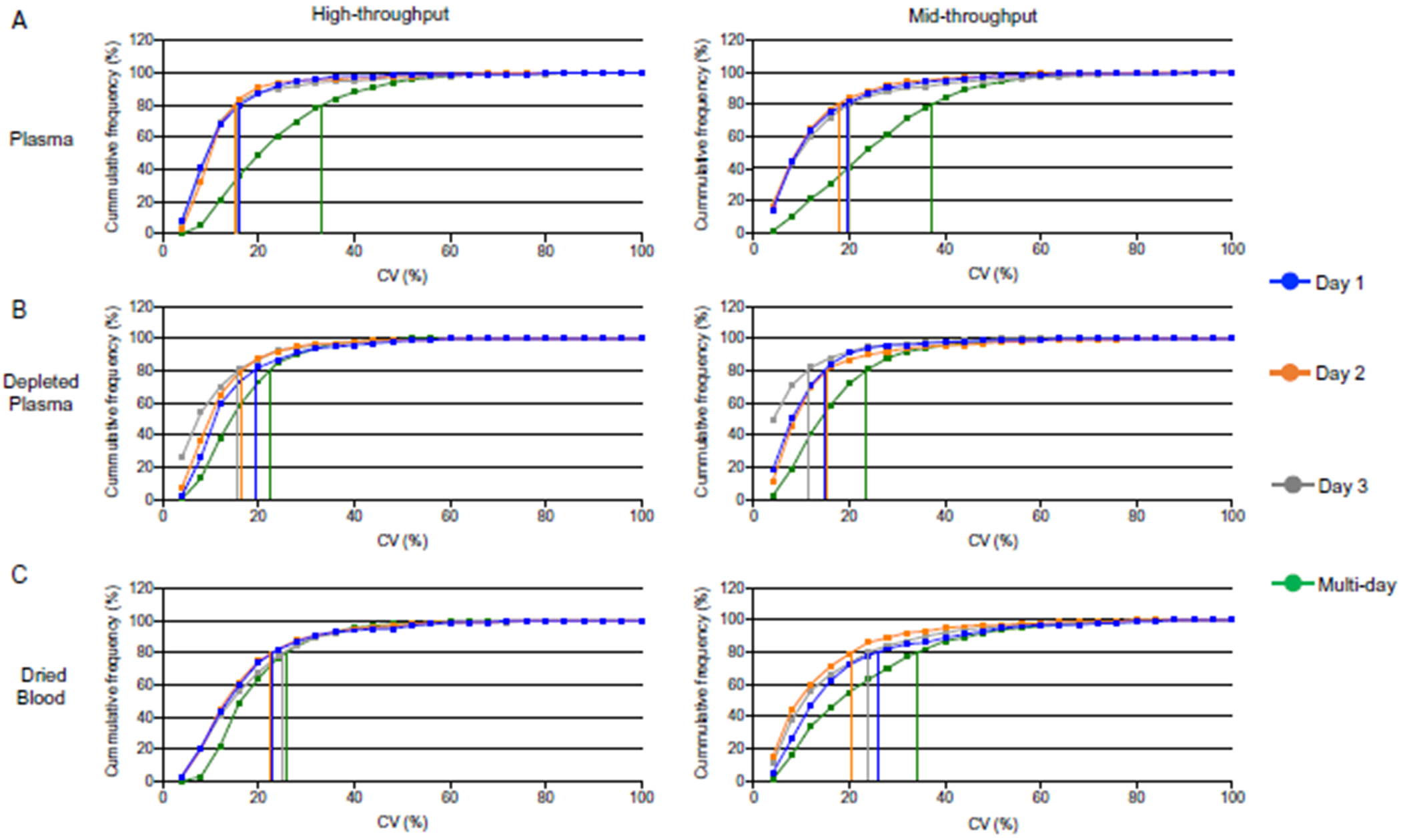
Evaluation of intra-day and inter-day precision in peptide quantification reproducibility. (A) Plasma, (B) depleted plasma and (C) dried blood C. Left panels: high-throughput. Right panels: mid-throughput. Vertical lines show the CV for 80% of peptides in each sample type and preparation day.

We performed a reproducibility study using the same blood biofluids as above. To determine the analytical precision, 5 replicate samples of each biofluid were digested each day for 3 consecutive days (total 15 replicates) and analyzed using the workflows. In the high-throughput method, cumulative frequency curves for the intra- and inter-day reproducibility showed most peptides identified on individual days displayed CV < 30% (Fig. 4, A to C, left). Comparing across all 3 days, plasma had the greatest drop in peptide intensity reproducibility with only 74% of peptides achieving a CV < 30% compared to 93% and 87% of peptides in depleted plasma and dried blood, respectively. In the mid-throughput platform, individual day peptide intensity reproducibility was high; >85% of the peptides identified had CV < 30% (Fig. 4, A to C, right). Interday data, again, showed plasma to be the least reliable with only 67% of identified peptides <30% CV compared to 90% for depleted plasma and 78% for dried blood.

**Fig. 5.**
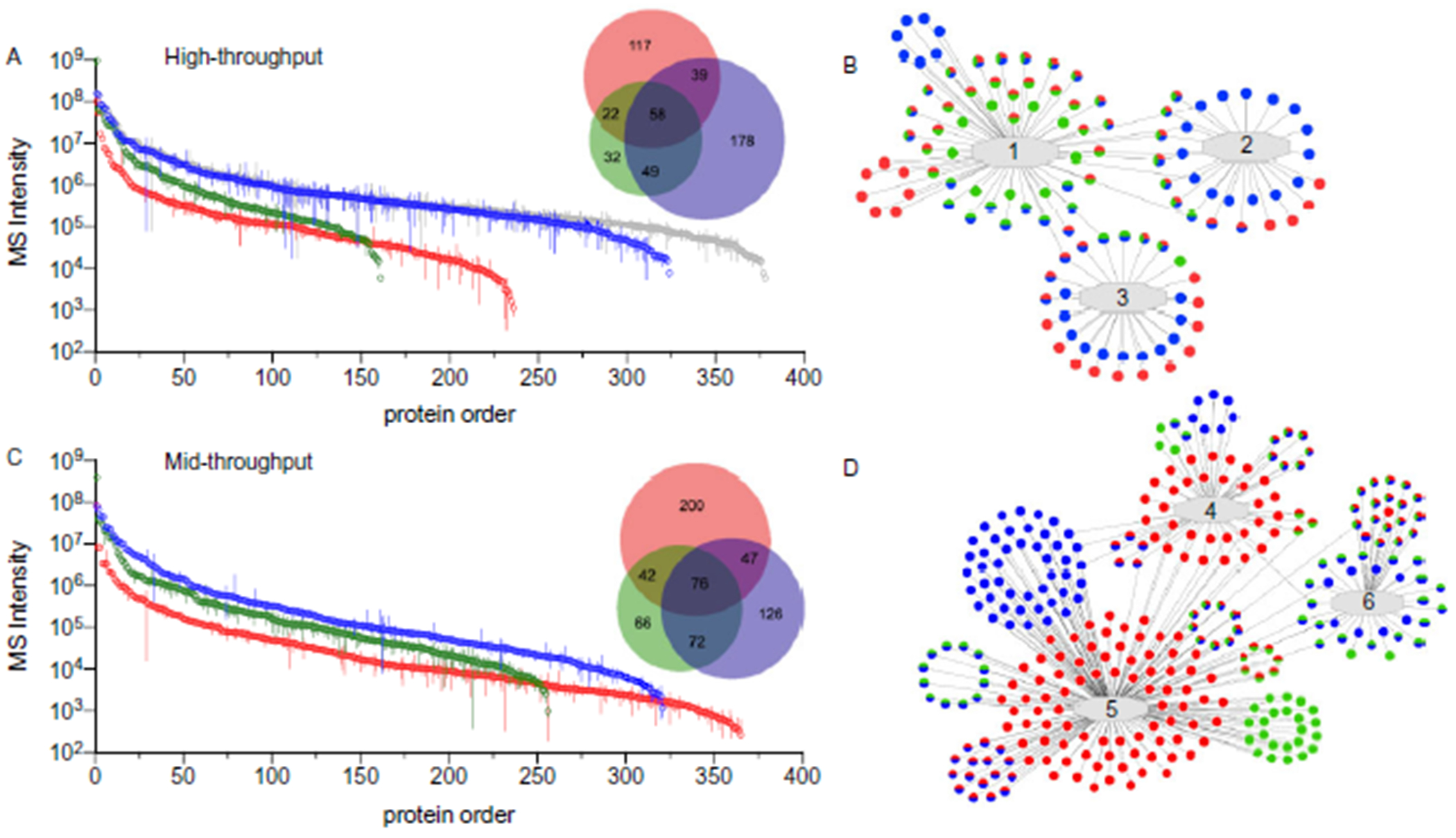
Comparisons of reliable proteomes in standardized workflows. Reliable protein observations are shown for naive plasma (green), depleted plasma (blue) and dried blood (red) in the high- (A) and mid-throughput (C) workflows. Proteins were ordered based on the multi-day mean intensity (error bars = SD). Venn diagrams match the stated color scheme. For comparison, proteins identified for naive and depleted plasma were combined (grey) indicating proteome coverage achieved by independent analysis of both biofluids. Representative examples of network analysis are shown for the high- (B) and mid- (D) throughputs. Networks: 1, adaptive immune response; 2, ERK1 and ERK2 cascade; 3, mitochondrial matrix;4, oxidoreductase activity; 5, intracellular non-membrane–bound organelles; 6, complement and coagulation cascade. Dots indicate identified proteins (colors match panels A and C). Gene names were omitted for space.

To compare the data sets produced from each biofluid and throughput method, we defined 4 levels of stringency, based on the precision of observations and linear response, to establish observable, reliable, reproducible, and quantifiable proteomes for each biofluid and workflow (Table 1). Comparison of the reliable proteomes (at least 3 observations/day) showed that depleted plasma had the largest number of proteins observed in the high-throughput method (324) while dried blood had the greatest number of proteins in the mid-throughput approach (365) (Fig. 5, A and C). While most biofluids had an increase in reliable identifications with the longer separation time, depleted plasma was essentially constant. As anticipated, each biofluid’s proteome had some overlap with the other proteomes and some unique components that were identified.

Since depleted plasma is derived from naive plasma and can easily be analyzed in the same study, we combined the individual analyses into a non-redundant list of general plasma identifications. The high-throughput reliable identifications from a combined naive and depleted plasma list was greater than the number of reliably identified proteins in any of the individual mid-throughput analyses (see online Supplemental Tables 16 to 19). A complete listing of all proteins identified, regardless of throughput method, have been provided for plasma (968, see online Supplemental Table 5), depleted plasma (893, see online Supplemental Table 10), dried blood (939, see online Supplemental Table 15) and combined naive and depleted plasma (1149, see online Supplemental Table 20).

As a further characterization of the various proteomes, a pathway analysis was performed on the reliably identified proteome for each biofluid (Fig. 5, B and D). In the high-throughput analysis, proteins from each proteome were observed in many of the networks. Naive plasma had an enrichment in detected proteins involved in innate immunity while dried blood and depleted plasma had a greater enrichment in mitochondrial matrix proteins. The mid-throughput analysis of dried blood showed enrichment of proteins involved in the oxidoreductase pathway while the complement and coagulation cascade were preferentially detected in the naive and depleted plasma analysis. Supplemental Tables 21 and 22 list the functional pathways identified in the bioinformatic analysis.

## Discussion

In this study we developed and optimized standardized workflows that could accommodate 3 distinct blood biofluids, assessed the quantifiability, and characterized the intra- and inter-day variability of the peptides and proteins detected. Previous studies have focused on deep proteome coverage utilizing elaborate fractionation or a long LC-MS run time, forgoing needs for increased throughput (17, 18). Other studies have emphasized reliability and quantifiability of proteins, focusing on a much more narrow aspect of the proteome (19). In our study we carried out linearity and reproducibility studies while comparing sample throughput in 3 distinct biofluids.

In the optimization of the standardized methods, we observed that some MS acquisition conditions resulted in greater numbers of peptide/protein identifications (IDs) but showed poor reproducibility of those observations. This was particularly apparent for the 15 Da window at 30k resolution in the high-throughput method (Fig 2, G). We found better precision with a wider precursor window (21 Da) and lower resolution (15k), which allowed for a greater sampling rate by the instrument producing a larger number of MS2 scans (shorter duty cycle). With these conditions we were able to balance the capture of total ion intensity with the number of observations across each chromatographic peak, resulting in a competitive number of IDs but higher reproducibility in the 21 Da 15k method (see Supplemental Fig. 2) compared to the other methods we evaluated. The combination of precision, intensity response, and number of observations for each chromatography method was factored in the selection of the best performing MS setting.

The implementation of one unified proteomic platform allows for flexibility in throughput and proteome coverage of 3 different biofluids. By defining each proteome based on the analytical performance and the inter- and intra-day reproducibility and linearity of each peptide and protein, at least in a healthy pool, a prospective user can tailor the workflow and biofluid to best meet the goals of a study. For example, transthyretin was characterized in the quantifiable proteome in all the analyses (5 and 4 plasma peptides; 34 and 4 depleted plasma peptides; 2 and 4 dried blood peptides, high- and mid-throughput, respectively). Alternatively, peroxiredoxin-6 was only detected in the quantifiable proteome of dried blood (11 peptides and 1 peptide in the high- and mid-throughput, respectively). Apolipoprotein L1 was quantifiable in both the naive and depleted plasma but in different workflows (0 and 6 plasma peptides, and 13 and 0 depleted plasma peptides in the high- and mid-throughput, respectively). These distinctions highlight the potential diversity of response any given analyte can have within the complex milieu of the different matrices and separation methods. When using a standardized platform, the anticipated needs of a study can guide how to balance the choice of sample type, depth, speed, and accuracy.

Collection of dried blood by a self-administered, minimally invasive finger prick device removes several barriers associated with the delivery of remote medical support (23, 24). We previously amended our original workflow for processing the 10 μL of blood absorbed and dried onto a Mitra^®^ microsampling device to conform with the plasma protocols (23). In our study reported here, we show that dried blood, while different in composition to naive and depleted plasma, can be analyzed with similar levels of reproducibility and quantifiability. Of note, our mid-throughput analysis of dried blood yielded the largest number of reliably identified proteins. Dried blood had the fewest pre-processing step of the biofluids evaluated, which may explain the greater number of reliable identifications. Our linearity experiments revealed the high-throughput assay was associated with reduced loading capacity compared to the mid-throughput assay (Fig. 3, A). A possible explanation is that background matrix resulting from hemolysis of red blood cells had a greater impact on the high-throughput assay in comparison to the mid-throughput assay. To fully address this, future interference studies need to be carried out.

The results from our precision study demonstrated that, with only modest adaptations, our plasma workflow can be used to reproducibly prepare samples derived from various clinical matrices (e.g., depleted plasma and dried blood). We observed that combining the naive and depleted plasma analyses from the high-throughput workflow produced a comparable number of reliable identifications compared to an individual mid-throughput analysis (Fig. 5, A). This is important since two 25-min runs on the Evosep One can be completed in 50 min or 25 min if using 2 MS instruments while a 60-min run on the Ultimate 3000 system requires 72 min to complete, including a 12-min blank run. Using the combined plasma approach, depending on the needs of the experiment, it might be possible to achieve a similar depth of analysis in approximately 33% to 70% of the instrument time depending on the number of instruments available.

## Conclusion

In summary, our streamlined process is associated with minimal hands-on time, fast turnaround, and excellent intra- and inter-day stability. The high-throughput platform supported reproducible detection of more than 74% of peptides in all matrices assessed. The mid-throughput platform supported reproducible detection of greater than 67% of peptides across all matrices. We observed that inter-day precision for depleted plasma was the most stable, with more than 90% of peptides reproducibly detected across days. Maximizing the commonality in the workflow between biofluids simplifies the approach and increases the likelihood for reproducibility across samples, studies, and institutions. Our broad vision for this platform is that it will help propel putative markers into clinical use.

## Supplementary Material

Supplemental_Information

## Figures and Tables

**Table 1.** Summary of proteins and peptides identified in different biofluids using the standardized workflow.

		Protein IDs				Peptide IDs			
		Observable	Reliable	Reproducible	Quantifiable	Observable	Reliable	Reproducible	Quantifiable
High-throughput		Total Obs^a^	>2 Obs/day	CV < 30%	CV < 30% *r*^2b^ > 0.8	Total Obs	>2 Obs/day	CV < 30%	CV < 30% *r*^2^ > 0.8

Naive plasma	Multi-day	240	161	133	98	1101	772	577	412

	mean /day	215 (3)	181 (5)	163 (4)	117 (4)	1021 (41)	903 (61)	857 (72)	574 (18)

Depleted plasma	Multi-day	681	324	202	117	2190	1216	1108	321

	mean/day	616 (13)	433 (31)	318 (18)	146 (8)	1996 (53)	1490 (102)	1376 (92)	353 (31)

Dried blood	Multi-day	365	236	189	121	823	530	466	320

	mean/day	350 (5)	280 (5)	223 (4)	129 (3)	775 (7)	618 (8)	546 (15)	341 (7)

Combined plasma	Multi-day	754	378	266	144	2676	1547	1390	887

	mean/day	674 (15)	486 (29)	383 (15)	169 (6)	2434 (74)	1875 (127)	1770 (115)	1003 (42)

Mid-throughput									

Naive plasma	Multi-day	572	256	168	152	2367	1416	1005	950

	mean/day	515 (45)	381 (82)	303 (59)	249 (38)	2184 (186)	381 (82)	1691 (327)	1435 (193)

Depleted plasma	Multi-day	462	321	281	198	2003	1618	1493	1051

	mean/day	438 (8)	373 (7)	333 (5)	221 (3)	1992 (25)	1765 (33)	1678 (38)	1108 (26)

Dried blood	Multi-day	564	365	279	185	1572	1087	874	554

	mean/day	518 (5)	426 (15)	353 (14)	209 (7)	1451 (5)	1238 (27)	1081 (68)	612 (18)

a Obs, observations.

b *r*^2^, square of the correlation coefficient.

## Nonstandard Abbreviations:

MS: mass spectrometry
DIA-MS: data independent acquisition-MS
TFE: 2,2,2-trifluoroethanol
LLOD: lower limit of detection
LLOQ: lower limit of quantification
