# Supplementary material for: Standardized Workflow for Precise Mid- and High-Throughput Proteomics of Blood Biofluids
Source: Clin Chem. Author manuscript; Available in PMC 2024 Jun 13. (PMC11175165; doi:10.1093/clinchem/hvab202)
Supplement: Supplemental_Information [file NIHMS1994904-supplement-Supplemental_Information.docx]

**Supplemental Information**

Methods

*Loading of Mitra tips*

Pool of human whole blood (with K_2_EDTA preservative) was obtained from Bioreclamation IVT (Chestertown, MD, US). All blood pools were stored at 4°C. Mitra® microsampling devices (Neoteryx, Torrance, CA, US) with 10 μL volumes were loaded with blood by dipping the tips into aliquoted 10 μL volume droplets of whole blood. Care was taken that the Mitra tips only touched the liquid surface and held at the droplet surface until fully colored red ensuring a complete fill. The filled Mitra tips were allowed to dry for at least 4 h at room temperature and stored until further processing in a closed container in the presence of a desiccant at -80°C.

*96-well Format Top 14 Abundant Protein Depletion for Plasma*

Plasma samples were depleted using the High Select Top 14 Abundant Protein Depletion Camel Antibody Resin (Thermo Fisher Scientific). On the day of depletion, anti-camel antibody-resin, which was stored at 4°C, was equilibrated to room temperature for 30 min mixing at 800 rpm. After equilibration, the anti-camel antibody-resin was vortexed vigorously and 300 μl was aliquoted into the wells of a 96 well plate (Nunc™ 96-Well Polypropylene DeepWell™ Storage Plates). 10 μl of plasma was diluted 1:10 with 100 mM NH_4_CO_3_ and added to wells containing depletion resin. To ensure homogenous mixing the plate was mixed at 800 rpm for 1 hour (hr). The unbound fraction was aspirated from the resin with 500 μl of 100 mM NH_4_CO_3_ and transferred to a filter plate (Nunc™ 96-Well Filter Plates). The depleted fraction was collected by gentle centrifugation (100 x *g* for 2 min) into a clean 96 well plate (Beckman Coulter, deep well titer plate polypropylene) and lyophilized.

*Automated Desalting*

Digested samples (300 μL) were mixed with 850 μL of 20 mL/L phosphoric acid, 1 mL/L formic acid. The desalting plate was activated with addition of 1 ml of 100 % methanol and equilibrated 3 times with the addition of 1 mL of 1 mL/L formic acid. 1150 μL of the acidified sample was applied to the plate and then washed with 3 applications of 1 mL/L formic acid. Peptide desalting was performed using Oasis 30 µm HLB 96-well Plate (Waters Co.) was adapted for the Beckman i7 automated workstation (1). The positive pressure apparatus (Amplius Positive Pressure ALP, Beckman Coulter) was mounted on the left side of the i7 workstation deck and the solid phase extraction pipetting steps (desalting) were carried out by i7 pipetting heads and controlled by Biomek Software (v5.1). For MeOH activation, filter pressure was set at 100 mBar and increased to 250 mBar over 1 min and 30 seconds. For washing and elution steps, the pressure started at 100 mBar and increased 50 mBar every 15 seconds to a final filter pressure of 550 mBar over 2 mins and 30 seconds. For each protocol a clamp pressure of 3000 mBar at the beginning and end. The sample was eluted with 0.5 mL of 500 mL/L acetonitrile, 1 mL/L formic acid and evaporated to dryness and stored at -80⁰C.  At the time of analysis, peptides were resuspended in a 1 mL/L formic acid solution.

*High- and Mid-throughput DIA LC-MS/MS*

DIA analysis was performed on an Orbitrap Exploris 480 (Thermo) instrument. For the high throughput workflow, the instrument was interfaced with an easy nano spray ion source coupled to an Evosep One. Peptides were separated on a preformed gradient (ranging from 50 – 350 mL/L organic phase) on a C18 column (3 µm beads, 100 µm ID, 8 cm long, Evosep) over the course of 21 min at a flow rate of 1000 nL/min. Source parameters included spray voltage at 2000 kV, capillary temp of 275°C and RF funnel level of 40. MS1 resolutions were set to 120,000 and AGC was set to 300% with ion transmission of 45 ms. Mass range of 350-1400 and AGC target value for fragment spectra of 300% were used. Peptide ions were fragmented at a normalized collision energy of 28%. Fragmented ions were detected across 50 DIA windows of 21 Da with an overlap of 1 Da (Adapted from (2)). MS2 resolution was set to 15,000 with an ion transmission time of 22 ms. All data was acquired in profile mode using positive polarity.

Mid-throughput DIA analysis was performed on an Orbitrap Exploris 480 (Thermo) instrument interfaced with a flex source coupled to an Ultimate 3000 ultra high-pressure chromatography system with 1 mL/L formic acid in water as mobile phase A and  1 mL/L formic acid in acetonitrile as mobile phase B. Peptides were separated on a linear gradient of 1-27% B organic phase for 45 min, 27-44% B for 15 min on a C18 column (15 cm with 300 µm ID, 3 µm Omega Polar C18 beads, and 100 Ǻ pore size, Phenomenex) over the course of total 60 mins at a flow rate of 9.5 μL/min. Between every sample the column was washed with a 10 min blank where the organic phase was increased to 98% and then re-equilibrated at 1% B for 2 min. Source parameters included; spray at 3000 kV, capillary temp of 300°C and an RF funnel level of 40. MS1 resolutions was set to 60,000 and AGC was set to “standard” with ion transmission of 100 ms. Mass range of 400-1000 and AGC target value for fragment spectra of 300% was used. Peptide ions were fragmented at a normalized collision energy of 30%. Fragmented ions were detected across 50 DIA non-overlapping 12 Da precursor windows. MS2 Resolution was set to 15,000 with an ion transmission time of 25 ms. All data is acquired in profile mode using positive polarity.

*DDA LC-MS/MS*

For DDA analysis was performed on an Orbitrap Exploris 480 (Thermo) instrument interfaced with an easy nano spray ion source coupled to an Evosep One. 500 ng of digested plasma was separated on a preformed gradient (ranging from 50 – 350 mL/L organic phase) on a C18 column (3 µm beads, 100 µm ID, 15 cm long, Evosep) over the course of 45 min at a flow rate of 1000 nL/min. Full MS resolution was set to 60,000, scan range was 350-1,250 m/z and AGC target was set to 300%. For MS2, resolution was 15,000, isolation width was 1.2 *m/z*, HCD energy was 28% and AGC target was set to 75%.

MS data and analysis has been deposited in the Panorama public repository (PXD ID: PXD024884) (3).

Bioinformatic Data Analysis

*DIA analysis*

LC-MS/MS data were visually inspected using XCalibur software (4.3.73.11). DIA MS raw files were converted to mzML, the raw intensity data for peptide fragments were extracted from DIA files using the OpenSWATH workflow (4) and searched against the Human Twin population plasma peptide assay library (5). Retention time predictions were made using the data from Biognosys iRT Standards spiked into each sample. Target and decoy peptides were then extracted, scored and analyzed using the mProphet algorithm to determine scoring cut‐offs consistent with 1% FDR (6). Peak group extraction data from each DIA file was combined using the ‘feature alignment’ script (7), which performs data alignment and modeling analysis across an experimental dataset. The total ion current (TIC) associated with the MS2 signal across the chromatogram was calculated for normalization using in-house software. This ‘MS2 Signal’ for each file was used to adjust the transition intensity of each peptide in a corresponding file. Normalized transition-level data was then processed using the mapDIA software (8) to perform quantitation at the peptide and protein level. All peptide and proteins identifications results can be found in the Supplemental Tables 1-20, accessible from the Panorama public repository (PXD ID: PXD024884) (3).

*DDA analysis*

DDA files were converted to mzXML and searched through the Trans Proteomic Pipeline (TPP) using three algorithms, 1) Comet, (9) 2) X!tandem! Native scoring (10) and 3) X!tandem! K-scoring (11) against a reviewed, Human canonical protein sequence database, downloaded from the Uniprot (12) database on May, 2018, containing 20341 target proteins and 20341 randomized decoy proteins. Precursor and fragment mass tolerance for each search algorithms was set to 20 ppm. Target-decoy modeling of peptide spectral matches was performed with PeptideProphet and the results searches were combined using the TPP InterProphet Parser (13). Peptides with a probability score of >95% from the entire experimental data set were imported into Skyline software (14) for quantification of precursor extracted ion intensities (XICs). Precursor XICs from each experimental file were extracted against the Skyline library, and peptide XICs with isotope dot product scores >0.8 were filtered for final analysis (15). Raw peptide intensities were used in the comparisons between experimental groups (50 mL/L TFE vs. 100 mL/L TFE or 4 h vs 16 h). The Skyline documents containing precursor XICs from each experimental file are available at Panorama (PXD ID: PXD024884) (3).

*Linearity and Reproducibility Analyses*

In the linearity analysis, lower limit of detection for a protein or peptide was determined by the lowest sample load where the protein or peptide was detected in at least 2 out of 3 replicates with a CV<20%. The lower limit of quantitation for a protein or peptide, was determined by the lowest sample load where the protein or peptide is detected in at least 2 out of 3 replicates with a CV<20% that is part of a minimum 3-point linear regression with an *r*^2^>0.8 and a target deviation>0.2. In the reproducibility analysis, CV for protein or peptide intensities were determined if there were at least 3 out of 5 observations on each day. This threshold was required for all 3 days to determine a multi-day CV. All peptide and proteins identifications results including linearity and reproducibility characterizations can be found in the Supplemental Tables 1-20 accessible from the Panorama public repository (PXD ID: PXD024884) (3).

*Functional enrichment analysis*

Functional pathway characterization and visualization was performed using ClueGO Ontology Analysis via PINE (Protein Interaction Network Extractor) (16,17). Visualization of significantly enriched terms in whole blood compared to native and depleted plasma in high- and mid-throughput methods. PINE consolidates protein interactions information from STRING and GeneMANIA to create a single, unified network (18,19). Category type analysis was performed in PINE to plot protein distribution across 3 categories: whole blood, native and depleted plasma from the high- and mid-throughput workflows. Venn diagrams were generated using Bio-venn (20). All proteins functional annotation results can be found in the Supplemental Tables 21-22, accessible from the Panorama public repository (PXD ID: PXD024884) (3).

References

1. Fu Q, Johnson CW, Wijayawardena BK, Kowalski MP, Kheradmand M, Van Eyk JE. A Plasma Sample Preparation for Mass Spectrometry using an Automated Workstation. J Vis Exp 2020;158: e59842.

2. Bekker-Jensen DB, Martínez-Val A, Steigerwald S, Rüther P, Fort KL, Arrey TN, et al. A Compact Quadrupole-Orbitrap Mass Spectrometer with FAIMS Interface Improves Proteome Coverage in Short LC Gradients. Mol Cell Proteomics 2020;19:716–29.

3. Panorama Public Repository. https://panoramaweb.org/im1z39.url (Accessed September 2021).

4. Wright I, Van Eyk JE. A Roadmap to Successful Clinical Proteomics. Clin Chem 2017;63:245–7.

5. Liu Y, Buil A, Collins BC, Gillet LCJ, Blum LC, Cheng L-Y, et al. Quantitative variability of 342 plasma proteins in a human twin population. Mol Syst Biol 2015;11:786.

6. Reiter L, Rinner O, Picotti P, Hüttenhain R, Beck M, Brusniak M-Y, et al. mProphet: automated data processing and statistical validation for large-scale SRM experiments. Nat Methods 2011;8:430–5.

7. Röst HL, Liu Y, D’Agostino G, Zanella M, Navarro P, Rosenberger G, et al. TRIC: an automated alignment strategy for reproducible protein quantification in targeted proteomics. Nat Methods 2016;13:777–83.

8. Teo G, Kim S, Tsou C-C, Collins B, Gingras A-C, Nesvizhskii AI, et al. mapDIA: Preprocessing and statistical analysis of quantitative proteomics data from data independent acquisition mass spectrometry. J Proteomics 2015;129:108–20.

9. Eng JK, Jahan TA, Hoopmann MR. Comet: an open-source MS/MS sequence database search tool. Proteomics 2013;13:22–4.

10. Craig R, Beavis RC. TANDEM: matching proteins with tandem mass spectra. Bioinformatics 2004;20:1466–7.

11. MacLean B, Eng JK, Beavis RC, McIntosh M. General framework for developing and evaluating database scoring algorithms using the TANDEM search engine. Bioinformatics 2006;22:2830–2.

12. UniProt Consortium. UniProt: a worldwide hub of protein knowledge. Nucleic Acids Res 2019;47:D506–15.

13. Keller A, Nesvizhskii AI, Kolker E, Aebersold R. Empirical statistical model to estimate the accuracy of peptide identifications made by MS/MS and database search. Anal Chem 2002;74:5383–92.

14. MacLean B, Tomazela DM, Shulman N, Chambers M, Finney GL, Frewen B, et al. Skyline: an open source document editor for creating and analyzing targeted proteomics experiments. Bioinformatics 2010;26:966–8.

15. Schilling B, Rardin MJ, MacLean BX, Zawadzka AM, Frewen BE, Cusack MP, et al. Platform-independent and label-free quantitation of proteomic data using MS1 extracted ion chromatograms in skyline: application to protein acetylation and phosphorylation. Mol Cell Proteomics 2012;11:202–14.

16. Bindea G, Mlecnik B, Hackl H, Charoentong P, Tosolini M, Kirilovsky A, et al. ClueGO: a Cytoscape plug-in to decipher functionally grouped gene ontology and pathway annotation networks. Bioinformatics 2009;25:1091–3.

17. Sundararaman N, Go J, Robinson AE, Mato JM, Lu SC, Van Eyk JE, et al. PINE: An Automation Tool to Extract and Visualize Protein-Centric Functional Networks. J Am Soc Mass Spectrom 2020;31:1410–21.

18. Szklarczyk D, Gable AL, Lyon D, Junge A, Wyder S, Huerta-Cepas J, et al. STRING v11: protein-protein association networks with increased coverage, supporting functional discovery in genome-wide experimental datasets. Nucleic Acids Res 2019;47:D607–13.

19. Montojo J, Zuberi K, Rodriguez H, Kazi F, Wright G, Donaldson SL, et al. GeneMANIA Cytoscape plugin: fast gene function predictions on the desktop. Bioinformatics 2010;26:2927–8.

20. Hulsen T, de Vlieg J, Alkema W. BioVenn - a web application for the comparison and visualization of biological lists using area-proportional Venn diagrams. BMC Genomics 2008;9:488.

Supplemental Tables

Supplemental Tables 1-5. Characterization of proteins and peptides detected in naive plasma using a high- and mid-throughput workflows.

Supplemental Tables 6-10. Characterization of proteins and peptides detected in depleted plasma using a high- and mid-throughput workflows.

Supplemental Tables 11-15. Characterization of proteins and peptides detected in dried blood using a high- and mid-throughput workflows.

Supplemental Tables 16-20. Characterization of non-redundant proteins and peptides detected in a combination of the naïve and depleted plasma analyses from the high-throughput workflow.

Supplemental Tables 21-22. Functional network assignments for proteins reliability detected in blood, plasma and depleted plasma in the high- and mid-throughput workflows.

All supplementary tables are accessible from the Panorama public repository (PXD ID: PXD024884) (3).

**Supplemental Fig. 1. Reproducibility of the automated i7 desalt (solid phase extraction) method.** The precision of total desalt workflow is comprised of both %CV from i7 workstation desalting and subsequent targeted LC-MS/MS. Stable isotope labeled internal standard (SIL) peptides spiked in digested human plasma were used to evaluate reproducibility of the method. The precision was determined from 18 wells/samples desalted with a HLB plate, processing 98 SIL internal standards in trypsin digested plasma in representative solid phase extraction experiment and %CVs for LC-MS/MS analysis and automated desalt processing were assessed with a highly multiplexed MRM assay (1). Briefly, tryptic plasma peptides and 98 internal standards were aliquoted into 18 wells and desalted by the i7 workstation. The desalted 98 SIL heavy peptides quantified by the MRM analysis with a Prominence UFLCXR HPLC system (Shimadzu, Japan) with a Waters Xbridge Peptide column coupled to a QTRAP^®^ 6500 with a Turbo V source. Analyst^®^ software (version 1.6.2 for the QTRAP 6500 was used to control the LC–MS system and for data acquisition. All MRM data were processed using MultiQuant™ 2.1 Software (SCIEX). The automated i7 desalt method demonstrated good reproducibility. The total %CV ranged from 4%-20% (with median 11% CV) for 247 transitions (representing 98 peptides and 53 proteins) after i7 desalting of SILs using targeted MRM analysis. The baseline of LC-MS errors in the MRM analysis were calculated from 7 repeated LC-MS injections. The identical 247 transitions showed %CV ranged from 1%-20% (with median 8%) for LCMS/MS.

**Supplemental Fig. 2. Optimization of DIA – MS for rapid, robust, deep and sensitive proteomic analysis of plasma.**

(**A**) Rank ordering of the mean intensities of peptides identified using the 21 Da isolation window at 30K (blue) or 15K (red) MS2 resolution. For comparison, the results of the 15Da window at 30K MS2 resolution (green) were included, as this method yielded the highest numbers in peptide identifications (Figure 2G). For each condition the error bars represent the standard deviation (n=3). The orange bands indicate high, mid and low intensity ranges (**B**) Comparison of the mean %CV between the three different MS2 settings at each of the peptide intensity ranges (n=100/range) (**C**) Plot of peptide intensity and CV for each MS2 resolution. The 21 Da 15K method showed a trend for more reproducible observations relative to intensity compared to the 21Da or 15 Da at 30K methods. (**D**) The average number of data points under the curve from 11 iRT standard peptides when analyzed using 25-min method and 8 different MS2 settings. The setting characterized in earlier panels are represented with the same colors. (**E**) The average number of data points under the curve from 11 iRT standard peptides when analyzed using 72-min method and 3 different MS2 settings.

**
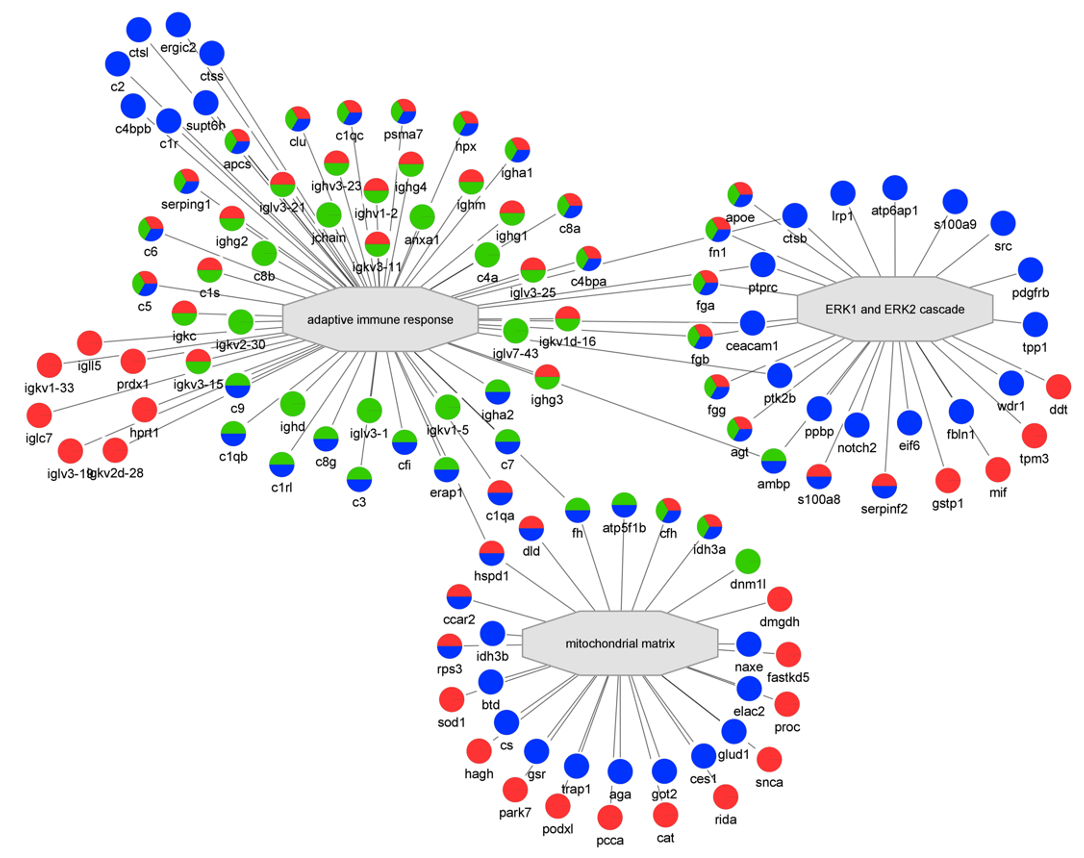
**

**Supplemental Fig. 3. Network analysis for high-throughput workflow.** An example of the PINE category type analysis is shown for reliably identified proteins (at least 3 observations on each of the 3 days) from the dried blood (red), naive plasma (green) and depleted plasma (blue). The central grey nodes denote term of enrichment. Supplemental Table 21 provides a complete listing of functional network assignments.

**
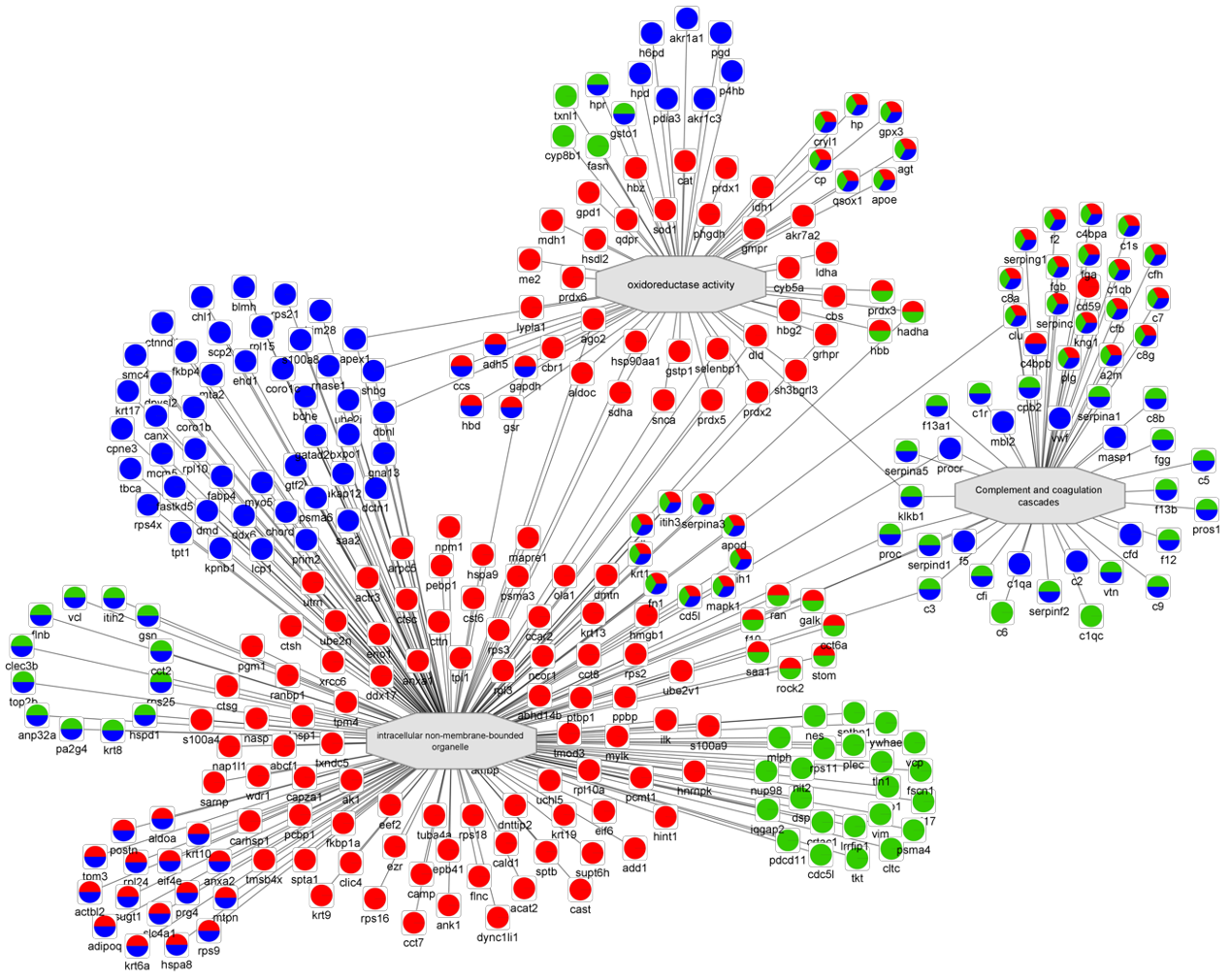
**

**Supplemental Fig. 4. Network analysis for mid-throughput workflow.** An example of the PINE category type analysis is shown for reliably identified proteins (at least 3 observations on each of the 3 days) from the dried blood (red), naive plasma (green) and depleted plasma (blue). The central grey nodes denote term of enrichment. Supplemental Table 22 provides a complete listing of functional network assignments.
